# Efficacy and safety of clopidogrel and aspirin in patients with chronic coronary syndrome: a systematic review and meta-analysis

**DOI:** 10.3389/fcvm.2026.1723015

**Published:** 2026-02-17

**Authors:** Xinzheng Wei, Ziqing Yang, Mingxu Chen, Hao Lu, Shengwei Hou

**Affiliations:** Department of Rehabilitation Medicine, Gongshu District People’s Hospital of Intergrated Traditional Chinese and Western Medicine, Hangzhou, Zhejiang, China

**Keywords:** aspirin, chronic coronary syndrome, clopidogrel, meta-analysis, stable coronary heart disease

## Abstract

**Objective:**

To systematically evaluate and compare the efficacy and safety of clopidogrel and aspirin, as well as their combination, in patients with chronic coronary syndrome (CCS), and to provide evidence-based support for long-term antiplatelet therapy in stable coronary artery disease.

**Methods:**

A comprehensive literature search was conducted in CNKI, WanFang, PubMed, Web of Science, and Embase databases from January 1990 to July 2025. Randomized controlled trials comparing clopidogrel and aspirin as monotherapy or in combination in patients with CCS were included. Relative risk (RR) or mean difference (MD) with 95% confidence intervals (CI) were pooled using fixed- or random-effects models according to heterogeneity. Publication bias and sensitivity analyses were performed where appropriate.

**Results:**

A total of 24 studies involving patients with chronic coronary syndrome were included. Compared with aspirin monotherapy, clopidogrel monotherapy was associated with a numerically lower incidence of myocardial infarction, although the difference was not statistically significant. No significant difference in the risk of serious bleeding events was observed between clopidogrel and aspirin. Combination therapy with clopidogrel and aspirin was associated with a higher treatment response rate and a tendency toward reduced angina frequency compared with aspirin alone; however, substantial heterogeneity was present, and the observed benefits were mainly confined to subjective or semi-quantitative outcomes. The incidence of common adverse reactions, including gastrointestinal discomfort, dizziness, and headache, was numerically lower in the combination therapy group, but these differences did not reach statistical significance.

**Conclusion:**

In patients with chronic coronary syndrome, clopidogrel represents a reasonable alternative to aspirin for long-term antiplatelet therapy, with comparable efficacy and safety. Combination therapy with clopidogrel and aspirin may offer modest improvements in symptom-related outcomes; however, these findings should be interpreted cautiously due to heterogeneity, potential publication bias, and the subjective nature of some endpoints. Individualized antiplatelet strategies based on ischemic and bleeding risk remain essential in stable coronary artery disease.

**Systematic Review Registration:**

https://www.crd.york.ac.uk/PROSPERO/view/CRD420251108327.

## Introduction

Chronic coronary syndrome (CCS), also referred to as stable coronary heart disease, represents a major clinical manifestation of coronary artery disease characterized by a stable atherosclerotic burden and long-term risk of ischemic events (1). Unlike acute coronary syndromes (ACS), CCS is dominated by chronic myocardial ischemia, recurrent angina symptoms, and progressive cardiovascular risk, requiring sustained secondary prevention strategies (2). With population aging and improved survival following acute events, the global prevalence of CCS continues to rise, imposing a substantial burden on healthcare systems (3–5).

The primary therapeutic goals in CCS include long-term symptom control, improvement of quality of life, and prevention of major adverse cardiovascular events. Antiplatelet therapy plays a central role in secondary prevention by inhibiting platelet aggregation and reducing thrombotic risk (6, 7).

Aspirin is a nonsteroidal anti-inflammatory drug (NSAID) that inhibits cyclooxygenase-1 (COX-1) activity, thereby reducing the synthesis of thromboxane A2 and inhibiting platelet aggregation (8). The antiplatelet effect of aspirin has been validated in numerous large-scale clinical trials and is widely used in the prevention and treatment of CAD (9, 10). However, the efficacy of aspirin therapy may be suboptimal in some patients, particularly those with aspirin resistance, where its antiplatelet effects are insufficient to effectively prevent ischemic events (11). Furthermore, long-term use of aspirin can lead to gastrointestinal side effects such as ulcers and indigestion, which may negatively impact patient adherence to treatment (12).

Clopidogrel, a P2Y12 receptor antagonist, works by inhibiting the P2Y12 receptor on platelets, thereby preventing ADP-induced platelet aggregation (13). Clopidogrel's mechanism of action differs from aspirin, and it has been shown to be more effective than aspirin in certain high-risk patient populations. For example, in patients with acute coronary syndrome or those who have undergone percutaneous coronary intervention (PCI), clopidogrel has demonstrated superior efficacy in reducing the incidence of myocardial infarction and stroke (14, 15). However, clopidogrel has its own drawbacks, such as a longer onset time and a higher bleeding risk (16).

In clinical practice, the combined use of clopidogrel and aspirin is widely applied in high-risk CAD patients, particularly in cases of acute coronary syndrome and post-stent implantation. This combination therapy offers dual inhibition of platelet function, further reducing the risk of cardiovascular events (17). However, the results of studies comparing the efficacy and safety of clopidogrel, aspirin, and their combination remain inconsistent. Although some studies suggest that combination therapy may be superior in certain clinical contexts, concerns about the potential bleeding risks and other adverse effects remain key considerations in clinical decision-making (17). Thus, finding an appropriate balance between efficacy and safety in treatment selection is an ongoing challenge in clinical practice.

Although several clinical studies have evaluated the efficacy of clopidogrel and aspirin, most are single trials and lack large-scale, systematic, comprehensive analyses. This is especially true for studies comparing the effects and safety of combined clopidogrel and aspirin treatment in different patient populations (18, 19). Meta-analysis, as a powerful statistical tool, can integrate data from multiple studies and provide more reliable conclusions. Therefore, the purpose of this study is to conduct a systematic review and meta-analysis to comprehensively evaluate the efficacy and safety of clopidogrel vs. aspirin in the treatment of CAD, particularly focusing on the comparison between monotherapy and combination therapy. This analysis will help explore their indications and risks in clinical treatment, offering guidance for clinicians to make more scientifically informed treatment decisions.

Therefore, this systematic review and meta-analysis aims to comprehensively assess the efficacy and safety of clopidogrel compared with aspirin, as well as their combination, specifically in patients with chronic coronary syndrome. By focusing on stable coronary populations, this study seeks to provide clearer evidence to guide long-term antiplatelet therapy in clinical practice.

## 2 Methods

### 2.1 Literature search strategy

This study followed the PRISMA 2020 guidelines for systematic reviews and meta-analyses, and the research questions and outcome indicators were defined prior to the literature search. Databases including CNKI, WanFang, PubMed, Web of Science, and Embase were searched. The search period was from January 1990 to July 2025, with the search limited to Chinese and English-language publications. Boolean operators were used to combine search terms related to aspirin, clopidogrel, coronary artery disease, and their synonyms. The search strategy was continuously refined according to the characteristics of each database. To minimize the risk of missing relevant studies, a “snowball” method was employed, where the references of retrieved studies were further searched for additional related articles. Registration No: PROSPERO: CRD420251108327.

### 2.2 Inclusion and exclusion criteria

Inclusion criteria:

1. Participants were adults diagnosed with chronic coronary syndrome or stable coronary heart disease, with disease duration generally exceeding 6 months.
2. Studies involving patients in the acute phase of coronary syndromes were excluded unless data for stable-phase patients could be independently extracted.
3. Prospective randomized controlled trials or cohort studies comparing clopidogrel and aspirin, either as monotherapy or combination therapy.
4. Studies reporting efficacy outcomes (such as myocardial infarction, MACCE, angina frequency) and/or safety outcomes (such as bleeding events).
5. The literature was published in Chinese or English, and within the search time frame.

Exclusion criteria:

1. Retrospective studies, reviews, case reports, conference abstracts, and animal studies.
2. Studies that did not compare clopidogrel and aspirin.
3. Studies that did not report efficacy or safety endpoints.
4. Duplicate publications or studies with incomplete data.

### 2.3 Literature screening and data collection

A rigorous literature screening process was followed to ensure the relevance and quality of the selected studies, with two researchers independently conducting the selection. In the preliminary screening phase, studies were excluded based on the title and abstract if they were deemed irrelevant or did not match the research topic. During the full-text screening phase, the full texts of potentially eligible studies were reviewed, and studies were further excluded based on predefined inclusion and exclusion criteria. These criteria included relevance to the research topic, whether the study involved human subjects, the completeness of the research methodology and results, and whether the study design was appropriate (such as randomized controlled trials, cohort studies, or case-control studies). Studies that did not meet these criteria were excluded. The data extracted from each study included participant details, study methodology, intervention measures, outcome indicators, and adverse reactions. If data were incomplete or unclear, efforts were made to contact the original authors to obtain supplementary information.

### 2.4 Literature quality assessment

In this study, the Cochrane Risk of Bias tool (RoB 2) was used to systematically evaluate the included RCTS. The evaluation included five areas: the randomization process, biased intervention delivery, missing outcome data, outcome measurement, and selective reporting. Each domain was scored as “low risk,” “some concern,” or “high risk,” and aggregated to derive an overall risk of bias rating for each study. Risk assessments were performed by two independent investigators, and disagreements were decided by discussion or arbitration by a third investigator.

### 2.5 Statistical analysis

For binary outcomes, the relative risk (RR) and its 95% confidence interval (95%CI) were used as the effect measure. For continuous variables, the mean difference (MD) and 95%CI were used. Heterogeneity was assessed using Cochran's *Q* test and the I^2^ statistic. When I^2^ > 50%, indicating high heterogeneity, a random-effects model (DerSimonian-Laird method) was used. Otherwise, a fixed-effects model was applied. Publication bias was visually assessed using a funnel plot and further tested using Egger's test (*P* < 0.1 suggests potential bias). When the number of included studies was more than 10, Egger's test was further used (*P* < 0.1 indicated the presence of bias). If the number of studies is less than 10, Egger's test has limited power and the results are only for reference. If necessary, subgroup analysis (based on region, follow-up time, sample size) and sensitivity analysis were conducted to test the robustness of the results. All statistical analyses were performed using R 3.4.3 software, and a *P*-value < 0.05 was considered statistically significant.

## 3 Results

### 3.1 Literature search results

**Figure 1:**
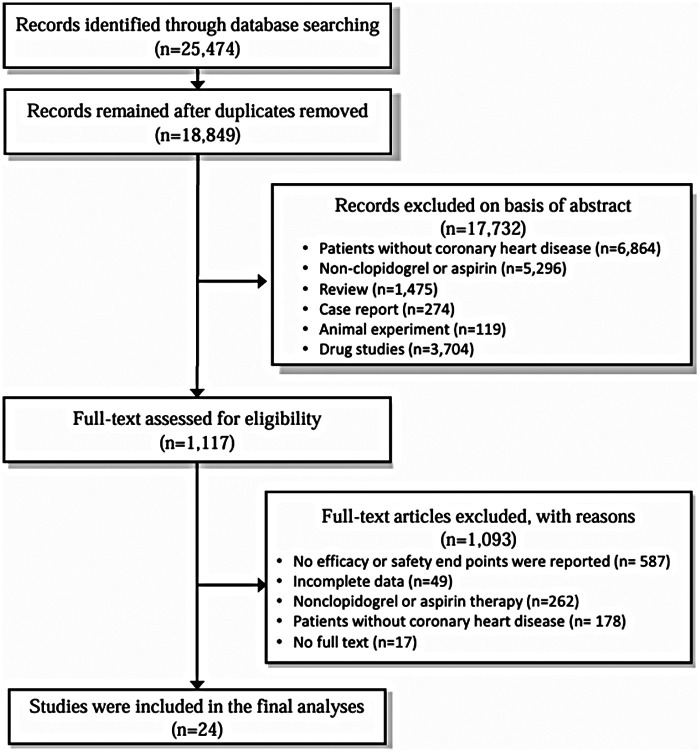
Literature selection flowchart.

A total of 25,474 articles were retrieved through the literature search. After removing duplicates, 18,849 records remained. Following title/abstract screening, 17,732 records were excluded. A further 1,117 studies were assessed through full-text review. From these, 587 studies that did not report efficacy or safety endpoints, 262 studies that did not involve clopidogrel or aspirin treatment, 178 studies with non-CAD patients, 49 studies with incomplete data, and 17 studies for which full texts could not be obtained were excluded. A total of 24 randomized controlled trials were included. The process for literature selection is shown in Figure 1.

### 3.2 Basic information of the included studies

A total of 24 studies were included in the analysis (Table 1), with study durations ranging from 2001 to 2025 and covering countries including China, the United States, South Korea, Japan, France, and the United Kingdom. All patients had chronic coronary syndrome, the duration of patients' illness exceeded one year. Intervention measures varied, with some studies comparing the efficacy and safety of clopidogrel monotherapy vs. aspirin monotherapy, while others evaluated the combination of clopidogrel and aspirin vs. aspirin monotherapy. The dose of clopidogrel was generally 75 mg per day, while aspirin doses ranged from 75 to 100 mg per day.

**Table 1:** Basic information of included studies.

Author	Year	Country	Course of Disease	Experimental group	Control group	Clopidogrel dose	Aspirin dose	outcome	Adverse effect
Deepak (20)	2001	America	>1 Years	Clopidogrel	Aspirin	75 mg/Day	100 mg/Day		bleeding
Bon (21)	2021	Korea	>1 Years	Clopidogrel	Aspirin	75 mg/Day	75-100 mg/Day	All-cause mortality, MI, Stroke, Rehospitalization	Gastrointestinal complications, BARC≥3, stent thrombosis
Gilles (22)	2016	France	>1 Years	Clopidogrel	Aspirin	75 mg/Day	75-100 mg/Day	MACE, MI, stroke	BARC≥3
Taek (23)	2016	Korea	>1 Years	Clopidogrel	Aspirin	75 mg/Day	75–100 mg/Day	MACE, MI, stroke	BARC≥3
Woodward (24)	2004	England	>1 Years	Clopidogrel	Aspirin	75 mg/Day	75 mg/Day	MI, FIB, CRP, D-Dimer, VWF, FVIII, t-PA, PV	Severe drug-related adverse reactions, bruising, lower respiratory tract infection, dyspnea
Zhuang (25)	2014	China	>1 Years	Clopidogrel	Aspirin	75 mg/Day	100 mg/Day	MI	Gastrointestinal complaints
Masahiro (26)	2020	Japan		Clopidogrel	Aspirin	75 mg/Day	75 mg/Day	all-cause mortality, MACE, MI, stroke	bleeding
Liu (27)	2025	China		Clopidogrel + Aspirin	Aspirin	75 mg/Day	100 mg/Day	Response rate of treatment, MI, MACE, LVEF, CO, SV,	Nausea, vomiting,Rash, headache
Xu (28)	2025	China	>3 Years	Clopidogrel + Aspirin	Aspirin	75 mg/Day	100 mg/Day	Response rate of treatment, Frequency of angina pectoris, TC, TG, LDL-C	Gastrointestinal complaints, Headache
Zhai (29)	2025	China	>4 Years	Clopidogrel + Aspirin	Aspirin	75 mg/Day	100 mg/Day	Response rate of treatment, Frequency of angina pectoris	Skin allergies, abnormal liver function, fatigue, NauseaVomiting
Fu (30)	2025	China	>6 Years	Clopidogrel + Aspirin	Aspirin	75 mg/Day	100 mg/Day	Response rate of treatment, MACE, TNF-α	
Liang (31)	2025	China	>2 Years	Clopidogrel + Aspirin	Aspirin	75 mg/Day	100 mg/Day	Response rate of treatment, TNF-α, IL-6, CRP	
Fu (32)	2025	China	>3 Years	Clopidogrel + Aspirin	Aspirin	75 mg/Day	100 mg/Day		
Wu (33)	2025	China	>6 Years	Clopidogrel + Aspirin	Aspirin	75 mg/Day	100 mg/Day	Response rate of treatment, MPV, PCT	Gastrointestinal bleeding, Headache, Rash,
Zhu (34)	2024	China	>8 Years	Clopidogrel + Aspirin	Aspirin	75 mg/Day	100 mg/Day	Response rate of treatment, LVEDD, LVESD, LVEF	Rash, Nausea, Vomiting, Headache, Gastrointestinal bleeding
Liu (35)	2024	China	>4 Years	Clopidogrel + Aspirin	Aspirin	75 mg/Day	100 mg/Day	Response rate of treatment, LVESD, LVEDD, LVEF, IL-6, TNF-α, CRP	
Hu (36)	2024	China		Clopidogrel + Aspirin	Aspirin	75 mg/Day	100 mg/Day	Response rate of treatment, Frequency of angina pectoris, SV, CI	NauseaVomiting, Rash, kidney injury
Han (37)	2024	China	>7 Years	Clopidogrel + Aspirin	Aspirin	75 mg/Day	100 mg/Day	Response rate of treatment, PAR, FIB, PT, Frequency of angina pectoris	Nausea, Vomiting, Gastrointestinal bleeding,
Yan (38)	2024	China	>3 Years	Clopidogrel + Aspirin	Aspirin	75 mg/Day	100 mg/Day	Response rate of treatment, PAR, FIB, PT, Frequency of angina pectoris	Rash, gastrointestinal reaction, tinnitus
Hu (39)	2024	China	>11 Years	Clopidogrel + Aspirin	Aspirin	75 mg/Day	100 mg/Day	Response rate of treatment, PT, APTT, PTA, CRP, LVEF, LVEDD, LVESD	Gastrointestinal bleeding, abnormal liver and kidney function, dyspepsia
Qiao (40)	2024	China	>6 Years	Clopidogrel + Aspirin	Aspirin	75 mg/Day	100 mg/Day	Response rate of treatment, MPV, LVEDD, LVESD, LVEF	Diarrhea, abdominal pain, dyspepsia, bleeding gums, Rash
Zhao (41)	2024	China		Clopidogrel + Aspirin	Aspirin	75 mg/Day	100 mg/Day	Response rate of treatment, IL-6, TNF-α, hs-CRP, APTT, PT	gastrointestinal reaction, swirl, Headache, Gastrointestinal bleeding,
Wang (42)	2024	China		Clopidogrel + Aspirin	Aspirin	75 mg/Day	100 mg/Day	Response rate of treatment	Nausea, Rash, Gastrointestinal bleeding, Headache
Deepak (43)	2006	America		Clopidogrel + Aspirin	Aspirin	75 mg/Day	100 mg/Day		bleeding

The primary outcomes reported in the included studies were (MACCE), myocardial infarction (MI), stroke, and all-cause mortality. Secondary outcomes included angina attack frequency, left ventricular ejection fraction (LVEF), lipid profiles, and inflammatory markers. Safety outcomes mainly included serious bleeding events (such as BARC ≥ 3), gastrointestinal reactions, skin rashes, headaches, and liver and kidney function abnormalities. Overall, all studies included clear efficacy and safety endpoints, making them suitable for meta-analysis.

### 3.3 Quality assessment of included studies

**Figure 2:**
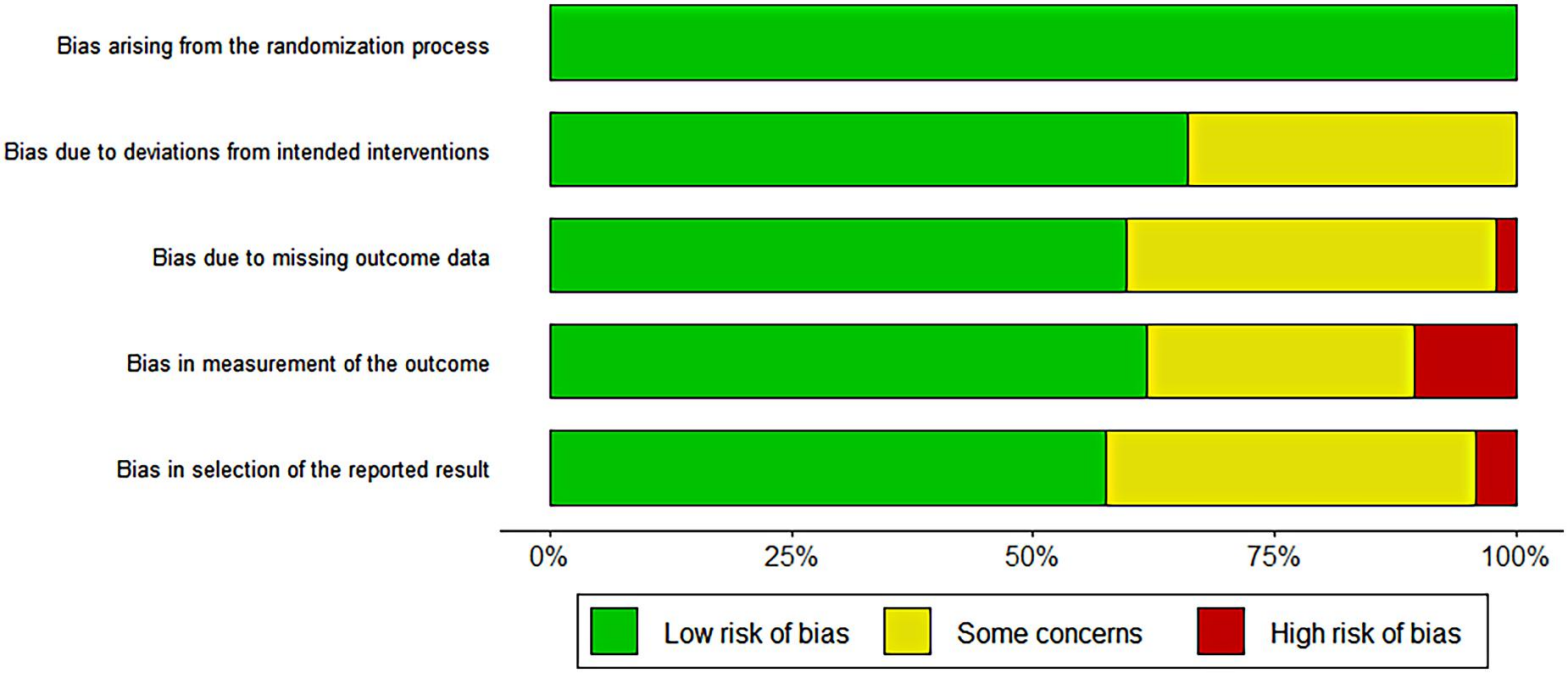
Cochrane risk of bias assessment results.

Cochrane Risk of bias tool was used to assess the risk of included studies. There were five evaluation dimensions: “randomization process”, “intended interventions”, “missing outcome data”, “measurement of the outcome” and “selection of the reported result”. The results of risk assessment showed that there were 15 low risk studies, 7 uncertain risk studies, and 2 high risk studies. In the dimension of bias during randomization, all studies were low risk, and in other aspects, some studies showed high or uncertain risk. The results are shown below. The high-risk studies were excluded and combined again in the subsequent meta-analysis, and the difference between the results before and after was not significant. Two high-risk studies were retained. The results are shown in Figure 2.

### 3.4 Efficacy of clopidogrel and aspirin treatment

**Figure 3:**
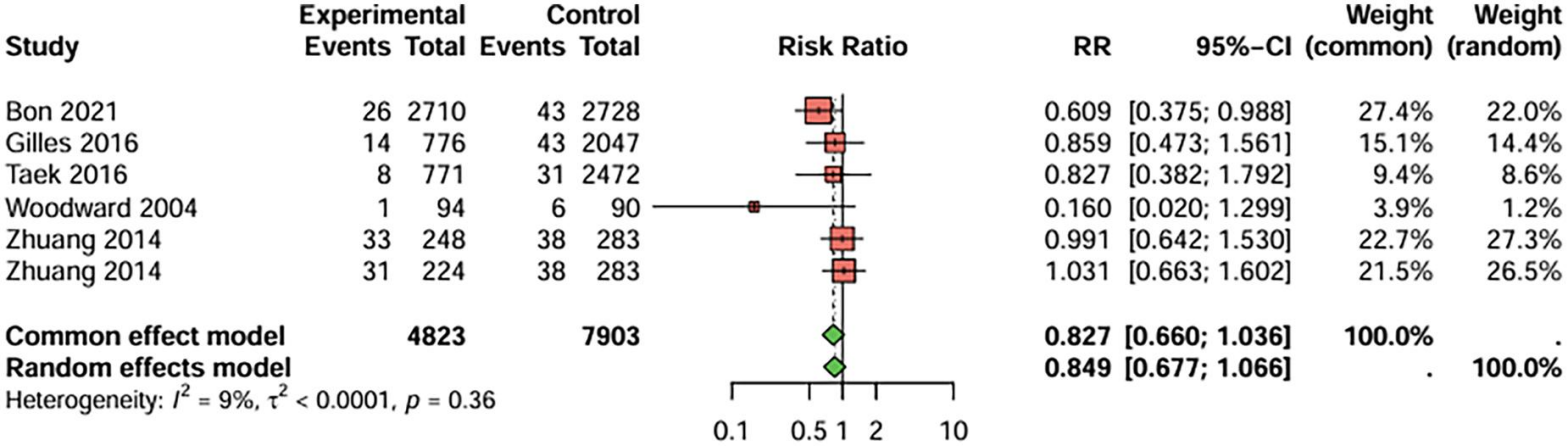
Meta-analysis result for myocardial infarction as the primary endpoint.

In studies comparing clopidogrel and aspirin monotherapy, six studies with myocardial infarction (MI) as the primary endpoint were included, the duration of treatment was more than 12 weeks (Figure 3). The combined analysis showed that the incidence of MI was lower in the clopidogrel group compared to the aspirin group, but the difference was not statistically significant. (RR = 0.827, 95%CI: 0.660–1.036). There was no significant heterogeneity between studies (I^2^ < 50%), and publication bias was not detected (t = −1.83, *P* = 0.143).

In studies comparing clopidogrel combined with aspirin to aspirin monotherapy, treatment efficacy was evaluated based on treatment success rate, frequency of angina attacks (per week). Some studies have reported the changes of prothrombin time (s) and thromboplastin time (s), but these indicators are mainly used to reflect the coagulation factor pathway function, and have nothing to do with the direct mechanism of antiplatelet drugs.

**Figure 4:**
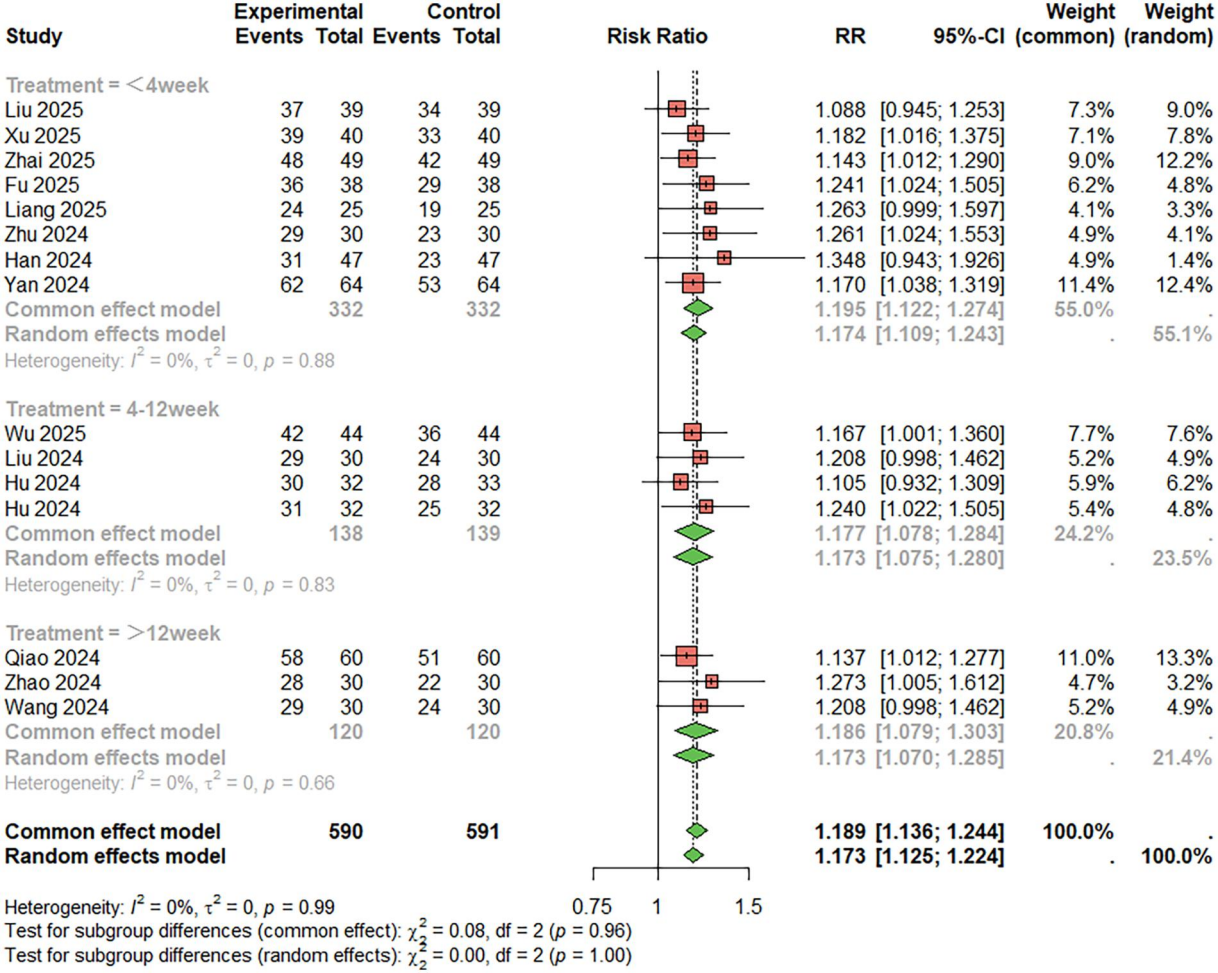
Meta-analysis result for treatment success rate.

For treatment success rate (Symptom improvement, patient-reported efficacy evaluation, and “effective/ineffective” grading of comprehensive subjective and objective indicators) (Figure 4), 15 studies were included. The combined analysis showed that the combination therapy group had a significantly higher success rate than the aspirin monotherapy group (RR = 1.189, 95%CI: 1.136–1.244), with low heterogeneity (I^2^ < 50%). The duration of treatment was less than 4 weeks, RR = 1.174, 95%CI: 1.109–1.243; Duration of treatment 4–12 weeks, RR = 1.173, 95%CI: 1.075–1.280; RR = 1.186, 95%CI: 1.079–1.303, treatment duration > 12 weeks. Egger's test indicated no publication bias (t = −1.63, *P* = 0.173) (Figure 4).

**Figure 5:**
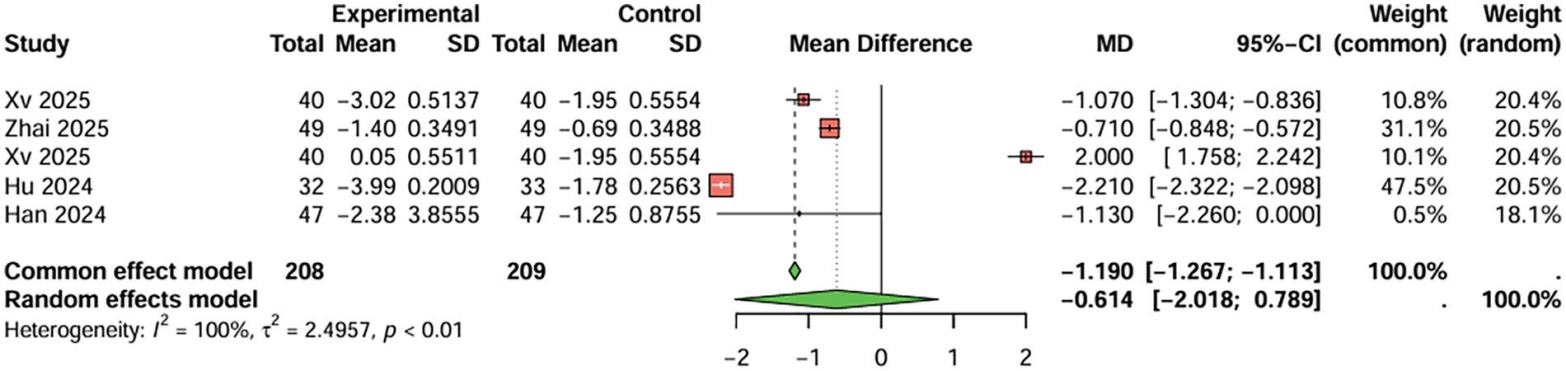
Meta-analysis result for frequency of angina attacks (per week).

Regarding the frequency of angina attacks, five studies were included. The combined result indicated that the combination therapy group had fewer angina attacks per week compared to the monotherapy group (MD = −0.614, 95%CI: −2.018–0.789 per week), with high heterogeneity (I^2^ > 50%). Publication bias was not significant (t = 0.89, *P* = 0.438) (Figure 5).

**Figure 6:**
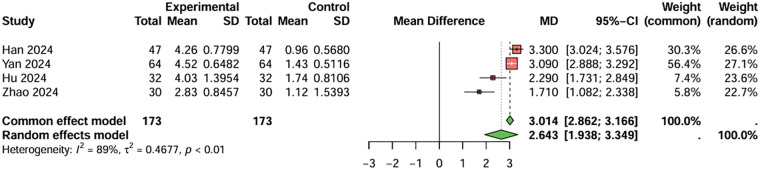
Meta-analysis result for prothrombin time (seconds).

**Figure 7:**
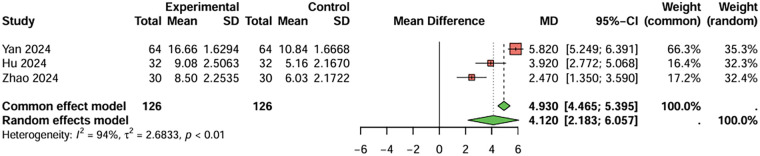
Meta-analysis result for activated partial thromboplastin time (seconds).

For prothrombin time, four studies were included, the duration of treatment was more than 12 weeks. The baseline prothrombin time for the combination therapy group before treatment was 9.688 s, and after treatment, it was 13.598 s. For the aspirin monotherapy group, baseline prothrombin time was 9.720 s, and after treatment, it was 11.033 s. The combined analysis showed that the prothrombin time in the combination therapy group was extended by 2.643 s (95%CI: 1.983–3.349) compared to the aspirin monotherapy group. After treatment, PT and APTT values were generally within or near the stated reference ranges (PT: 11–13 s; APTT: 31–43 s), although baseline values were below these ranges and PT in the combination therapy group was slightly above the upper limit. The heterogeneity test showed no significant heterogeneity (I^2^ < 50%), and therefore a fixed-effects model was used. Egger's test showed no significant publication bias (t = −2.49, *P* = 0.129) (Figure 6). For activated partial thromboplastin time, four studies were included, the duration of treatment was more than 12 weeks. The baseline activated partial thromboplastin time for the combination therapy group before treatment was 27.430 s, and after treatment, it was 38.843 s. For the aspirin monotherapy group, baseline activated partial thromboplastin time was 27.550 s, and after treatment, it was 34.893 s. The combined analysis showed that the activated partial thromboplastin time in the combination therapy group was extended by 4.120 s (95%CI: 2.183–6.057) compared to the aspirin monotherapy group. The heterogeneity test showed no significant heterogeneity (I^2^ < 50%), and therefore a fixed-effects model was used. Egger's test showed no significant publication bias (t = −2.70, *P* = 0.112) (Figure 7). In conclusion, the pooled analysis of this study showed that although PT and APTT changed after treatment, they were generally within or near the stated reference ranges, suggesting limited clinical significance.

### 3.5 Safety analysis of clopidogrel and aspirin treatment

**Figure 8:**
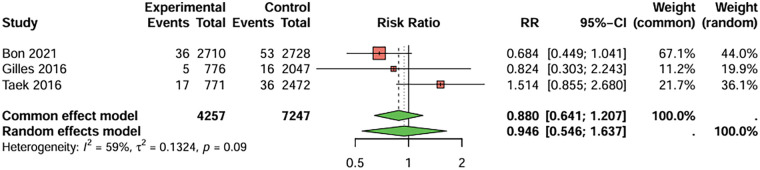
Meta-analysis result for serious bleeding (BARC ≥ 3).

In studies directly comparing clopidogrel and aspirin, three studies with BARC ≥ 3 bleeding as the primary endpoint were included, the duration of treatment was more than 1 years. The combined analysis showed no statistically significant difference in the risk of serious bleeding events between the two groups (RR = 0.946, 95%CI: 0.546–1.637), indicating that clopidogrel did not increase the occurrence of serious bleeding events (Figure 8). There was no significant heterogeneity between studies (I^2^ < 50%), and Egger's test did not detect significant publication bias (t = −0.34, *P* = 0.794).

For the safety analysis comparing clopidogrel combined with aspirin to aspirin monotherapy, primary outcomes included gastrointestinal reactions, dizziness, headaches, nausea, vomiting, and skin rashes.

**Figure 9:**
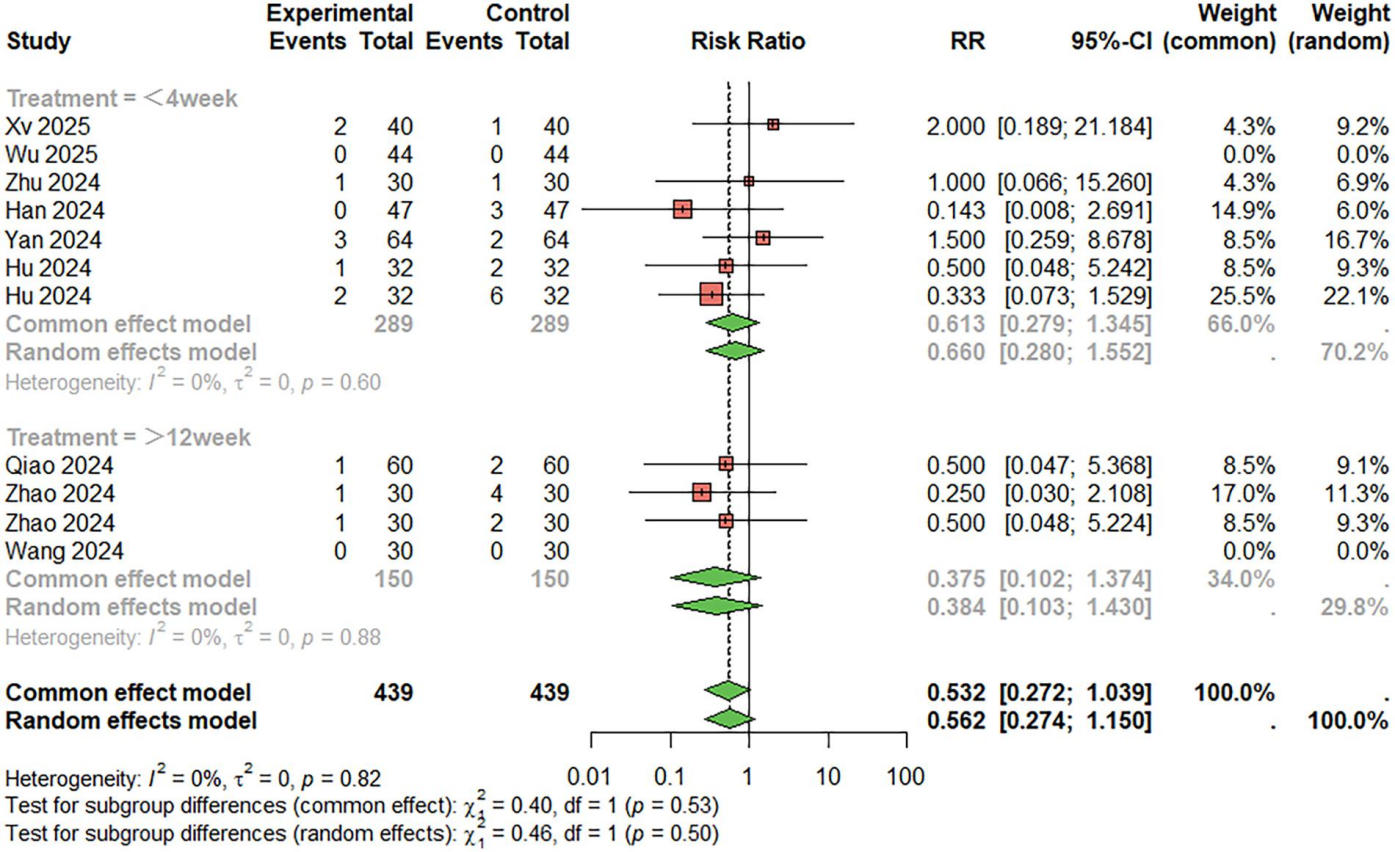
Meta-analysis result for gastrointestinal reactions.

Regarding the incidence of gastrointestinal reactions (Figure 9), 11 studies were included. The combined analysis showed that the combination therapy group had a lower incidence of gastrointestinal reactions than the aspirin monotherapy group (RR = 0.532, 95%CI: 0.272–1.039), although the difference was not statistically significant. There was no significant heterogeneity between studies (I^2^ < 50%). When the treatment duration was less than 4 weeks, RR = 0.613, 95%CI: 0.279–1.345; When the treatment duration was more than 12 weeks, RR = 0.375, 95%CI: 0.102–1.374. Egger's test did not suggest significant publication bias (t = −0.11, *P* = 0.915).

**Figure 10:**
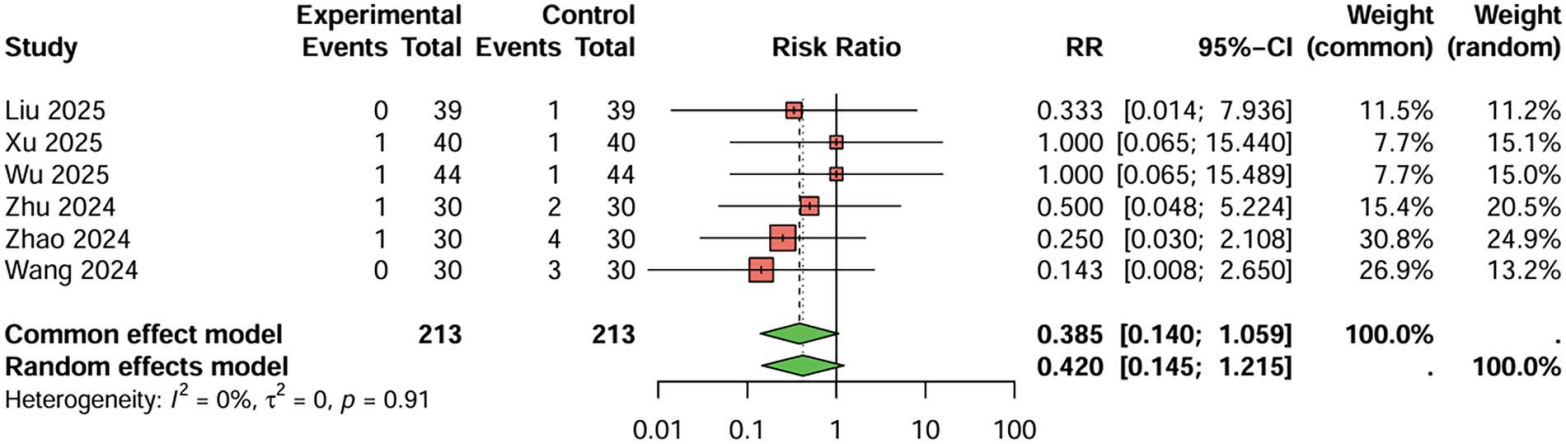
Meta-analysis result for dizziness and headaches.

For dizziness and headaches (Figure 10), six studies were included, the duration of treatment was more than 12 weeks. The combined analysis showed a lower incidence of dizziness and headaches in the combination therapy group compared to the monotherapy group (RR = 0.385, 95%CI: 0.140–1.059), but this difference did not reach statistical significance. The heterogeneity between studies was low (I^2^ < 50%), and Egger's test did not suggest significant publication bias (t = 0.23, *P* = 0.828).

**Figure 11:**
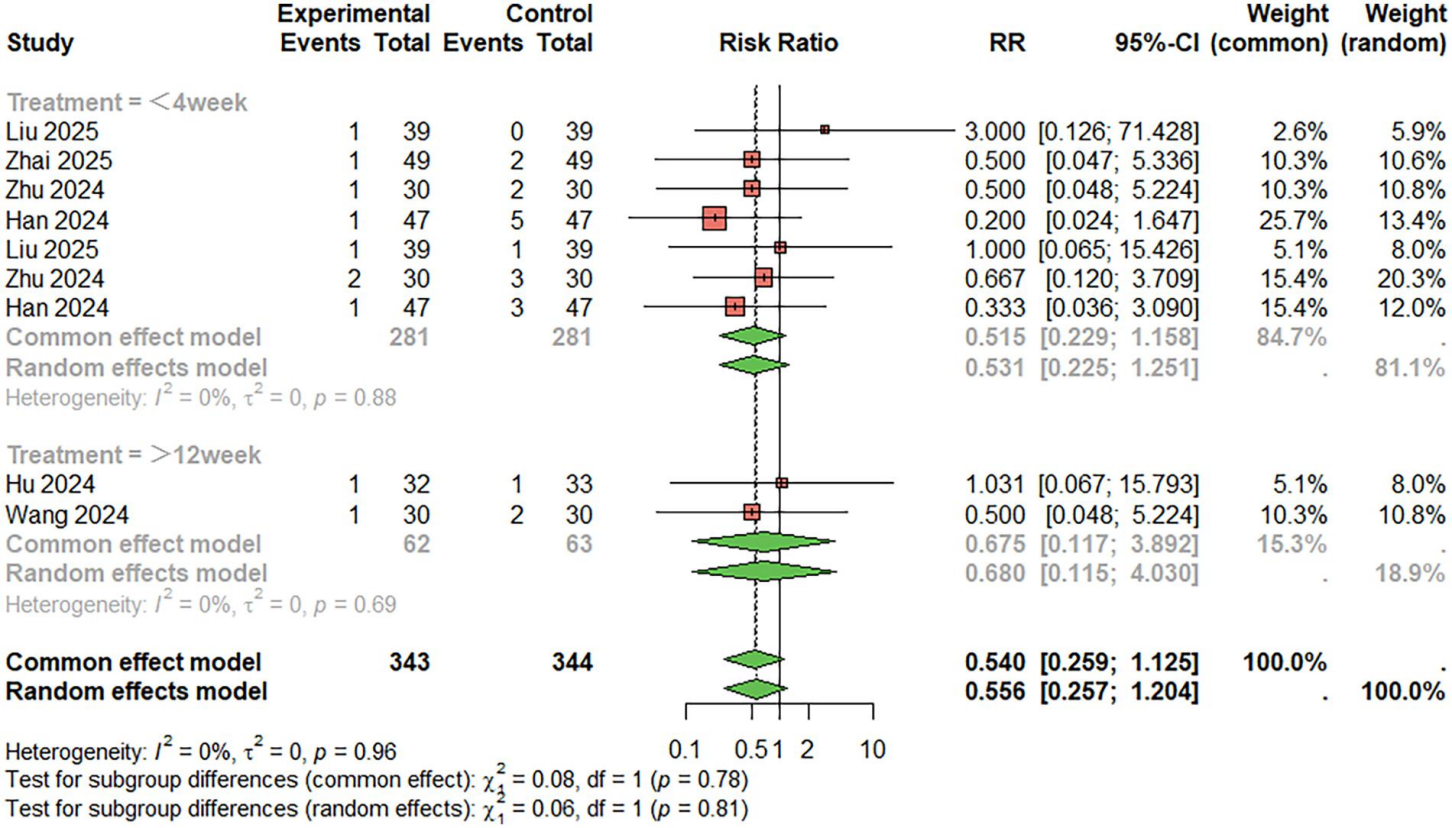
Meta-analysis result for nausea and vomiting.

For nausea and vomiting (Figure 11), nine studies were included. The combined analysis showed a lower incidence of nausea and vomiting in the combination therapy group compared to the monotherapy group (RR = 0.540, 95%CI: 0.259–1.125), but the difference was not statistically significant. The heterogeneity between studies was low (I^2^ < 50%). When the treatment duration was less than 4 weeks, RR = 0.515, 95%CI: 0.229–1.158; When the treatment duration was more than 12 weeks, RR = 0.675, 95%CI: 0.117–3.892. Egger's test did not suggest significant publication bias (t = 1.74, *P* = 0.125).

**Figure 12:**
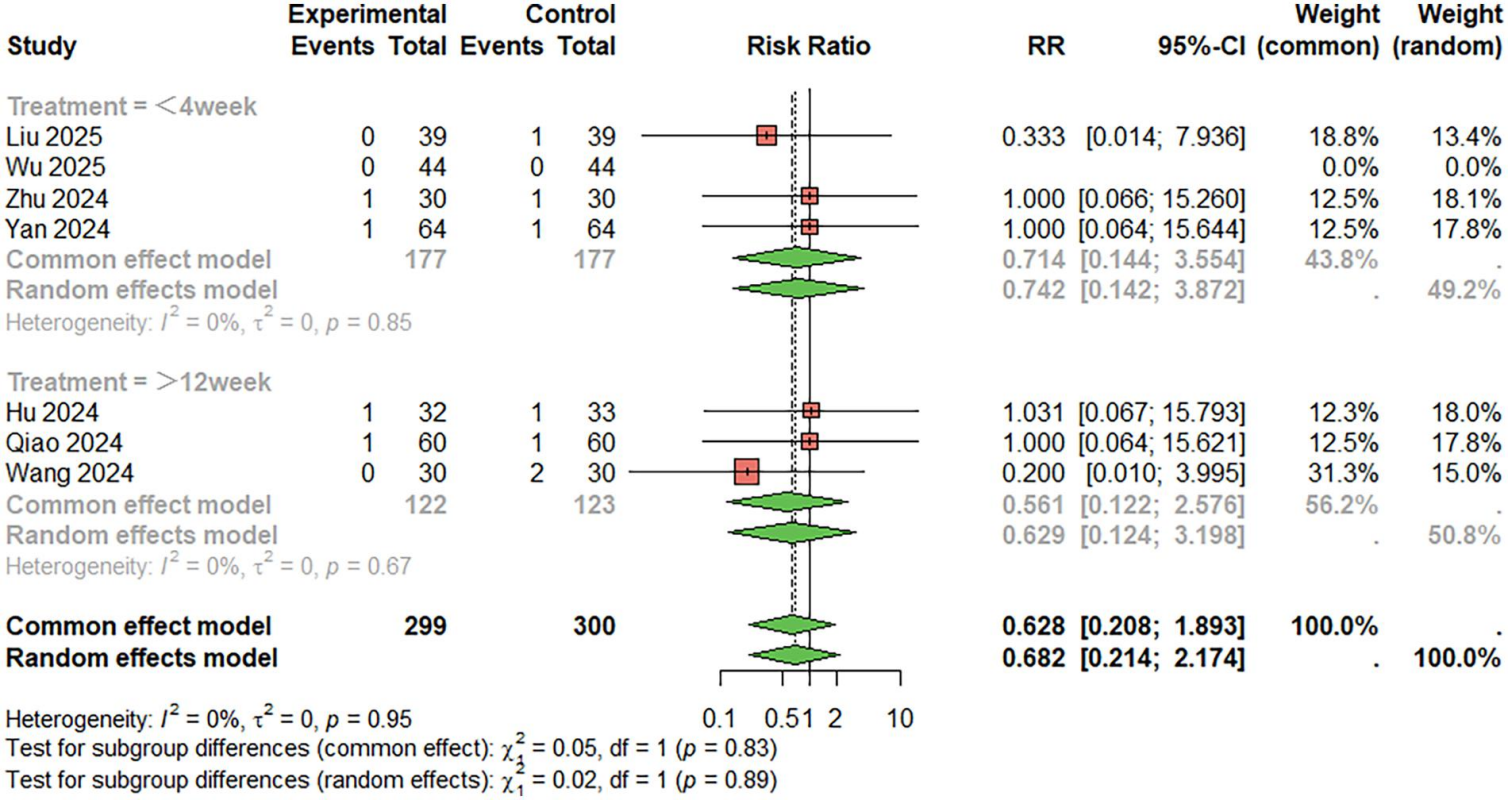
Meta-analysis result for skin rashes.

For skin rashes (Figure 12), seven studies were included. The combined analysis showed that the incidence of skin rashes was lower in the combination therapy group compared to the monotherapy group (RR = 0.628, 95%CI: 0.208–1.893), although the difference was not statistically significant. There was no significant heterogeneity between studies (I^2^ < 50%). When the treatment duration was less than 4 weeks, RR = 0.714, 95%CI: 0.144–3.554; When the treatment duration was more than 12 weeks, RR = 0.561, 95%CI: 0.22–2.576. Egger's test did not suggest significant publication bias (t = 1.24, *P* = 0.269).

Overall, there were no significant differences in the risk of serious bleeding events between clopidogrel and aspirin monotherapy, and the combination therapy group showed a lower incidence of gastrointestinal reactions, dizziness, nausea, vomiting, and skin rashes compared to the aspirin monotherapy group. However, these differences did not reach statistical significance.

## 4 Discussion

### 4.1 Main findings

This systematic review and meta-analysis synthesized evidence from 24 studies to evaluate the efficacy and safety of clopidogrel and aspirin in patients with chronic coronary syndrome (CCS). The main findings can be summarized as follows. First, clopidogrel monotherapy showed a numerically lower incidence of myocardial infarction compared with aspirin monotherapy, although this difference did not reach statistical significance. Importantly, no significant increase in serious bleeding events was observed, suggesting that clopidogrel provides a comparable safety profile to aspirin in stable coronary populations.

Second, dual antiplatelet therapy with clopidogrel and aspirin was associated with improved symptom-related outcomes, including a higher overall treatment response rate and a tendency toward reduced angina frequency compared with aspirin alone. However, these benefits were primarily observed in subjective or semi-quantitative endpoints, and substantial heterogeneity was present in some analyses. Therefore, while combination therapy appears to offer potential symptomatic advantages, these findings should be interpreted cautiously, particularly in the absence of consistent effects on hard clinical endpoints.

Overall, the results indicate that clopidogrel represents a reasonable alternative to aspirin for long-term antiplatelet therapy in CCS, whereas the incremental benefit of combination therapy appears modest and largely confined to symptom-related outcomes.

### 4.2 Comparison with previous studies

The findings of this meta-analysis are broadly consistent with previous large-scale trials and meta-analyses evaluating antiplatelet therapy, such as the CAPRIE and CURE studies, which demonstrated the efficacy of clopidogrel, either alone or in combination with aspirin, in reducing ischemic events (44, 45). However, it is important to note that these landmark trials primarily focused on patients with acute coronary syndromes or recent revascularization, and their results cannot be directly extrapolated to stable CCS populations.

By specifically restricting the analysis to patients with chronic coronary syndrome, the present study extends existing evidence to a clinically important but less well-studied population. Compared with acute or high-risk settings, the observed benefits of clopidogrel or dual therapy in CCS appear attenuated, emphasizing long-term tolerability and safety rather than pronounced reductions in major ischemic events (46).

On the other hand, although previous studies have highlighted the efficacy of clopidogrel, this study also found considerable heterogeneity between the results of different studies, particularly in long-term follow-up patients, where differences in treatment effects and adverse reactions were more pronounced. This variation could be attributed to the diversity of baseline characteristics among the patients, as well as differences in drug regimens, dosing schedules, and routes of administration across studies. Therefore, the challenge remains in how to tailor the best treatment strategy for individual patients based on their specific characteristics. In this study, combination therapy showed a modest advantage over aspirin alone in reducing the frequency of angina episodes, but there was high heterogeneity in the pooled analysis (I^2^ > 50%). The possible reasons for this are as follows: (1) The assessment methods of “angina episode” are inconsistent among studies, some rely on patient self-report, some based on clinical records or electrocardiographic evidence; (2) The follow-up time varied greatly, ranging from several weeks to several years. Different follow-up time may affect the recording of angina pectoris attack frequency. (3) There were differences in the baseline characteristics of the patients, such as the proportion of hypertension, diabetes or previous PCI history, which may affect the occurrence of angina pectoris. (4) Although the intervention program and drug dosage are generally consistent, there may be differences in medication compliance and the application of combined drugs (such as *β*-blockers and nitrates). All of the above factors may lead to inconsistent results between studies, thereby increasing the heterogeneity of the analysis. Therefore, the findings need to be interpreted with caution and cannot be simply generalized to all patient populations.

### 4.3 Possible mechanisms of action

The differing pharmacological mechanisms of clopidogrel and aspirin provide a biological rationale for the observed findings. Aspirin irreversibly inhibits cyclooxygenase-1, thereby reducing thromboxane A₂ synthesis and platelet aggregation, whereas clopidogrel selectively blocks the platelet P2Y12 receptor, inhibiting ADP-mediated platelet activation. When used in combination, these agents exert complementary antiplatelet effects, resulting in more potent platelet inhibition than either drug alone (47).

In this analysis, slight prolongations of prothrombin time (PT) and activated partial thromboplastin time (APTT) were observed in some studies involving combination therapy; however, these values generally remained within normal reference ranges. Given that aspirin and clopidogrel primarily act on platelet function rather than the coagulation cascade, PT and APTT should be considered exploratory rather than definitive indicators of antiplatelet safety. The modest changes observed are more likely attributable to interindividual variability, concomitant medications, or laboratory conditions rather than direct pharmacological effects.

Importantly, the bleeding risk associated with dual antiplatelet therapy is predominantly mediated through enhanced platelet inhibition rather than alterations in coagulation factor activity (48, 49). Therefore, normal or near-normal coagulation parameters do not preclude an increased bleeding tendency, highlighting the need for careful clinical risk assessment when considering combination therapy.

### 4.4 Clinical implications

The findings of this study have several clinical implications. First, for patients with CCS who are intolerant to aspirin or exhibit inadequate response, clopidogrel may serve as an effective and safe alternative for long-term antiplatelet therapy. The comparable efficacy and bleeding risk profile observed in this analysis support its use in appropriately selected patients.

Second, while combination therapy with clopidogrel and aspirin may improve symptom-related outcomes, its routine use in stable CCS should be approached cautiously. The observed benefits were largely confined to subjective endpoints and were not accompanied by consistent reductions in major adverse cardiovascular events. Therefore, dual antiplatelet therapy should not be interpreted as a replacement for guideline-recommended strategies in stable coronary disease but may be considered on an individualized basis, particularly in patients with persistent symptoms and low bleeding risk (50).

Special attention should be given to elderly patients and those with a history of gastrointestinal disease, renal dysfunction, or elevated bleeding risk, in whom the potential benefits of intensified antiplatelet therapy must be carefully balanced against safety concerns.

### 4.5 Publication bias

The potential impact of publication bias should be carefully considered. Although Egger's test did not consistently indicate significant publication bias for most outcomes, the presence of bias cannot be completely excluded, particularly for symptom-based and subjective endpoints.

Several factors may contribute to publication bias in the included literature. First, a substantial proportion of the studies were small-scale trials conducted in single centers, in which positive findings-especially improvements in symptom-related outcomes such as treatment response rate or angina frequency-are more likely to be published than neutral or negative results. Second, many studies originated from regional or local journals, where selective reporting of favorable outcomes may be more common, and negative findings are less frequently submitted or accepted for publication.

Third, the lack of standardized definitions for outcomes such as “treatment response rate” increases the risk of selective outcome reporting, as investigators may preferentially report endpoints showing statistically significant differences. In contrast, hard clinical endpoints such as myocardial infarction or major bleeding events are less susceptible to reporting bias, as they are more objectively defined and often mandated outcomes in randomized trials.

Finally, language restrictions to English and Chinese publications may have introduced additional bias, as studies published in other languages or unpublished data from clinical registries were not captured. Taken together, these factors suggest that publication bias may have influenced the magnitude of the observed effects, particularly for subjective outcomes, and further underscore the need for cautious interpretation of the pooled results.

### 4.6 Limitations

Several limitations of this study should be acknowledged. First, a substantial proportion of the included studies were small, single-center trials, many of which originated from China, potentially limiting the generalizability of the findings. Second, symptom-based outcomes such as treatment response rate lacked standardized definitions across studies and were inherently subjective, introducing heterogeneity and potential reporting bias.

Third, although publication bias was formally assessed, the possibility of selective reporting—particularly for positive symptom-related outcomes—cannot be entirely excluded. In addition, patient-level data were unavailable, precluding subgroup analyses based on genetic factors (such as CYP2C19 polymorphisms), bleeding risk scores, or comorbid conditions, which are increasingly recognized as important determinants of antiplatelet response.

Finally, some surrogate endpoints included in the analysis, such as PT and APTT, are not ideal indicators of antiplatelet efficacy or safety and should be interpreted as exploratory findings rather than clinically decisive evidence.

### 4.7 Future research directions

Future research should prioritize large-scale, well-designed randomized controlled trials focusing on patients with chronic coronary syndrome and using clearly defined, objective clinical endpoints. Particular attention should be given to identifying subgroups that may derive greater benefit from alternative or intensified antiplatelet strategies, such as patients with diabetes, prior revascularization, or high ischemic risk but low bleeding risk.

In addition, emerging P2Y12 inhibitors, including ticagrelor and prasugrel, warrant further investigation in stable coronary populations. Advances in pharmacogenomics may also facilitate more personalized antiplatelet therapy, optimizing efficacy while minimizing adverse events (50).

## 5 Conclusion

This systematic review and meta-analysis evaluated the efficacy and safety of clopidogrel and aspirin, as well as their combination, in patients with chronic coronary syndrome. The findings indicate that clopidogrel monotherapy provides comparable efficacy and safety to aspirin for long-term antiplatelet treatment in stable coronary populations, with no significant increase in serious bleeding events. Clopidogrel may therefore serve as a reasonable alternative for patients who are intolerant to or inadequately protected by aspirin.

Combination therapy with clopidogrel and aspirin was associated with modest improvements in symptom-related outcomes, including treatment response rate and angina frequency. However, these benefits were primarily observed in subjective or semi-quantitative endpoints and were accompanied by substantial heterogeneity and potential publication bias. No consistent reduction in major adverse cardiovascular events was demonstrated, and the observed changes in coagulation parameters remained within normal ranges, suggesting limited clinical relevance.

Overall, the results support the use of individualized antiplatelet strategies in patients with chronic coronary syndrome, balancing ischemic risk, bleeding risk, and symptom burden. Routine use of dual antiplatelet therapy in stable coronary disease cannot be recommended based solely on the current evidence. Further large-scale, high-quality randomized controlled trials with standardized, objective clinical endpoints are needed to clarify the role of combination antiplatelet therapy in this population.
